# Conservative Treatment Without Any Intervention Compared With Other Therapeutic Strategies for Symptomatic Patent Ductus Arteriosus in Extremely Preterm Infants: A Nationwide Cohort Study in Korea

**DOI:** 10.3389/fped.2021.729329

**Published:** 2021-10-06

**Authors:** Jeonghee Shin, Jin A Lee, Sohee Oh, Eun Hee Lee, Byung Min Choi

**Affiliations:** ^1^Department of Pediatrics, Korea University College of Medicine, Seoul, South Korea; ^2^Department of Pediatrics, Seoul National University Boramae Hospital, Seoul, South Korea; ^3^Medical Research Collaborating Center, Seoul National University Boramae Hospital, Seoul, South Korea

**Keywords:** symptomatic patent ductus arteriosus, conservative treatment, pharmacological intervention, surgical intervention, extremely preterm infants

## Abstract

**Objective:** Although symptomatic treatment is the most preferred treatment strategy for proven symptomatic patent ductus arteriosus (PDA), a considerable number of infants only received conservative treatment without any pharmacological or surgical interventions in the lower gestational age and lower birth weight group in Korea. We compared in-hospital outcomes of infants treated conservatively without any intervention and those of infants managed by other therapeutic strategies in extremely preterm infants with symptomatic PDA.

**Methods:** A prospectively collected cohort study for 2,303 infants with gestational ages <28 weeks from the Korean Neonatal Network database. These infants were classified into four groups according to the presence of PDA-related symptoms and therapeutic treatment strategy: prophylactic treatment group, pre-symptomatic treatment (PST) group, symptomatic treatment (ST) group, and conservative treatment (CT) without any intervention group.

**Results:** In multivariable logistic regression analysis, the risk of death was significantly decreased in the PST group (adjusted odds ratio [aOR] = 0.507; 95% confidence interval [CI] 0.311–0.826) and ST group (aOR = 0.349; 95% CI: 0.230–0.529) compared with the CT group. However, the risk of composite outcome of severe bronchopulmonary dysplasia or death had not increased in the PST group and ST group. Neonatal death due to pulmonary hemorrhage or neurological disease was significantly higher in the CT group than in the PST group or ST group.

**Conclusion:** In extremely preterm infants, who are at highest risk of PDA-related morbidities and mortality, even less interventional approach for PDA can be allowed; the rescued pharmacological or surgical interventions are necessary if they met the criteria for hemodynamically significant PDA.

## Introduction

Many neonatologists have attempted pharmacological or surgical interventions to promote closure of patent ductus arteriosus (PDA) under the assumption that early closure can decrease the possibility of PDA-related morbidities. However, these interventions carry risks of short- and long-term adverse effects and complications (1–3). In addition, they might not reduce the risk of mortality and morbidities such as necrotizing enterocolitis, bronchopulmonary dysplasia (BPD), and neurodevelopmental impairment (4–8). Therefore, there have been growing calls for either abandoning routine interventions and/or more selective individualized interventions for PDA closure (9–18). Moreover, recent studies on adoption of conservative treatment for PDA at several centers (19–24) have demonstrated that less intervention strategies do not appear to lead to increased morbidity or mortality in preterm infants, although they have hemodynamically significant PDA (hsPDA) (20, 25, 26).

In our previous report (27) from Korean Neonatal Network (KNN) registry, nearly half of very low birth weight (VLBW) infants with symptomatic PDA received various therapeutic strategies for PDA. Although symptomatic treatment for PDA was the most preferred treatment strategy, a considerable number of infants only received conservative treatment without any pharmacological or surgical interventions in lower gestational age and lower birth weight group in Korea. Therefore, many neonatologists are concerned that conservative treatment alone without using any pharmacological or surgical interventions as a rescue treatment for symptomatic PDA may increase the risk for neonatal adverse outcomes from prolonged exposure of hsPDA.

The objective of this study is to compare in-hospital outcomes of infants treated conservatively without any intervention and those of infants managed by other therapeutic strategies in extremely preterm infants with symptomatic PDA using data from KNN registry.

## Methods

### Data Source and Study Cohort

This was a cohort study using prospectively collected data for 2,303 VLBW infants with gestational ages <28 weeks born between 2013 and 2015 from the KNN database. Clinical data were collected from 60 participating hospitals by local staff using a standardized electronic case report form in the KNN database and analyzed prospectively for the purposes of this study. VLBW infants admitted to neonatal intensive care units (NICUs) at birth or transferred from other hospitals within 28 days of life to KNN participating hospitals were registered to the KNN registry. Data included baseline characteristics, PDA treatments, morbidities, and mortality from birth until discharge or until the age of 1 year old (corrected age). The KNN registry was approved by the institutional review board at each participating hospital. Informed consent to collect and use clinical data and outcome of VLBW infants was obtained from parents at enrollment by NICUs participating in the KNN.

We excluded 332 infants due to any major congenital or chromosomal anomaly (63 infants), missing or mismatched information about PDA treatment strategy and details of pharmacological or surgical treatment of PDA (13 infants), unknown policy for PDA treatment (34 infants), hospital admission for more than 1 year (4 infants), transfer to other hospital (84 infants), and death within 3 days after birth (134 infants). Since PDA-related death and treatment strategies might not be properly evaluated within 3 days of birth, deaths within 3 days after birth were excluded from this study.

A total of 1,971 VLBW infants were finally included in the analysis. Among these infants, we identified cases whose PDA closed spontaneously or had minimal ductal shunting before any signs and symptoms were attributable to PDA. We also identified those who did not receive any treatment due to the absence of PDA-related symptoms (defined as “no PDA group”). The remaining patients were classified into four groups according to the presence of PDA-related symptoms and therapeutic treatment strategy: (1) prophylactic treatment (PT) group, (2) pre-symptomatic treatment (PST) group, (3) symptomatic treatment (ST) group, and (4) conservative treatment (CT) without medication or surgical intervention group.

### Definitions of PDA Treatment Strategies

PDA treatment strategies are defined according to the KNN manual of operation as follows: (1) PT is used for those who have no PDA-related clinical symptoms or diagnostic abnormalities to reduce the incidence of intracerebral hemorrhage or PDA requiring treatment; (2) PST is used for those whose PDA is confirmed by echocardiography, but treatment is started only after meeting diagnostic criteria such as BNP or after positive findings in echocardiography before showing clinical signs of PDA; (3) ST is used for those who show clinical symptoms of PDA with hemodynamically significant left to right shunt by echocardiography; and (4) CT is defined as conservative and supportive treatment without any pharmacological or surgical intervention for those with clinical symptoms of PDA with hemodynamically significant shunt by echocardiography. Presence of clinical symptoms attributable to PDA was defined when there were two out of the following five symptoms caused by PDA: (1) systolic or continuous murmur; (2) bounding pulse or hyperactive precordial pulsation; (3) hypotension; (4) respiratory difficulty; and (5) pulmonary edema or cardiomegaly (cardiothoracic ratio > 60%) on chest radiograph.

### Study Outcomes

Baseline characteristics included gestational age, birth weight, gender, multiple gestation, mode of delivery, use of antenatal steroids, small for gestational age (SGA), pregnancy-induced hypertension (PIH), histological chorioamnionitis, resuscitation on delivery room (DR), 5-min Apgar score, CRIB-II (an update of the clinical risk index for babies) score, surfactant use, and early-onset sepsis. Gestational age was obtained from obstetric medical records. SGA was defined when birth weight was less than the tenth percentile for gestational age. PIH was defined if there was any maternal diagnosis of eclampsia or preeclampsia. DR resuscitation was defined when cardiac compression was done or medication was administered in the DR. CRIB II score was calculated from the database considering birth weight, gestational age, body temperature, base excess, and sex of the baby. Use of surfactant was defined when it was used for prophylaxis or treatment of respiratory distress syndrome of newborn. Early sepsis was defined as a positive blood culture <7 days from birth.

Outcome variables included death before discharge, neonatal mortality, duration of invasive ventilation, duration of respiratory support, discharge with respiratory support, BPD defined as the need for supplemental oxygen and/or positive pressure at 36 weeks postmenstrual age (PMA) or discharge, severe BPD defined as need for ≥30% oxygen and/or positive pressure at 36 weeks PMA or discharge, NEC ≥ stage 2, intraventricular hemorrhage (IVH) ≥ Grade 3, cystic periventricular leukomalacia (PVL), retinopathy of prematurity (ROP) with treatment, hospital day, and secondary PDA ligation.

Regarding the cause of death, we used data inputted by the physician in each unit according to Korean Standard Classification of Diseases based on the International Classification of Diseases (10th revision, Clinical Modification). It had the following mutually exclusive categories: cardiorespiratory causes, including respiratory distress syndrome, pulmonary hemorrhage, air leak syndrome, BPD, and other cardiorespiratory diseases; neurological causes, including hypoxic–ischemic encephalopathy or asphyxia, severe IVH and its sequelae, and other neurological diseases; infection causes, including congenital infection, acquired infection, and other infectious diseases; gastrointestinal causes, including NEC or spontaneous intestinal perforation and other gastrointestinal diseases; and others, including trauma or accident, inborn errors of metabolism, multisystemic failures of unknown etiology, and other abnormalities.

### Statistical Analysis

Continuous variables were expressed as median [interquartile range]. Categorical variables were expressed as number and proportion. Baseline characteristics, in-hospital outcomes, and causes of death were compared according to PDA treatment strategies. Kruskal–Wallis test was used for continuous variables. Chi-square test or Fisher's exact test were used for comparing categorical variables. For multiple comparisons, Bonferroni correction was applied. Multivariable logistic regression models (variables with *p* < 0.01 in univariable analysis, gestational age, multiple gestation, antenatal steroids, histologic chorioamnionitis, and DR resuscitation) were performed to determine associations of PDA treatment strategies with in-hospital outcomes. Birth weight was not used in this statistical analysis due to its high correlation with gestational age. Adjusted odds ratios (aORs) and their 95% confidence intervals (CIs) were obtained for each outcome variable comparing CT group with treatment groups. All statistical analyses were conducted using IBM SPSS Statistics version 20 (IBM Corp., Amarok NY, USA). Statistical significance was considered when two-sided *P*-value was < 0.05.

## Results

**Figure 1:**
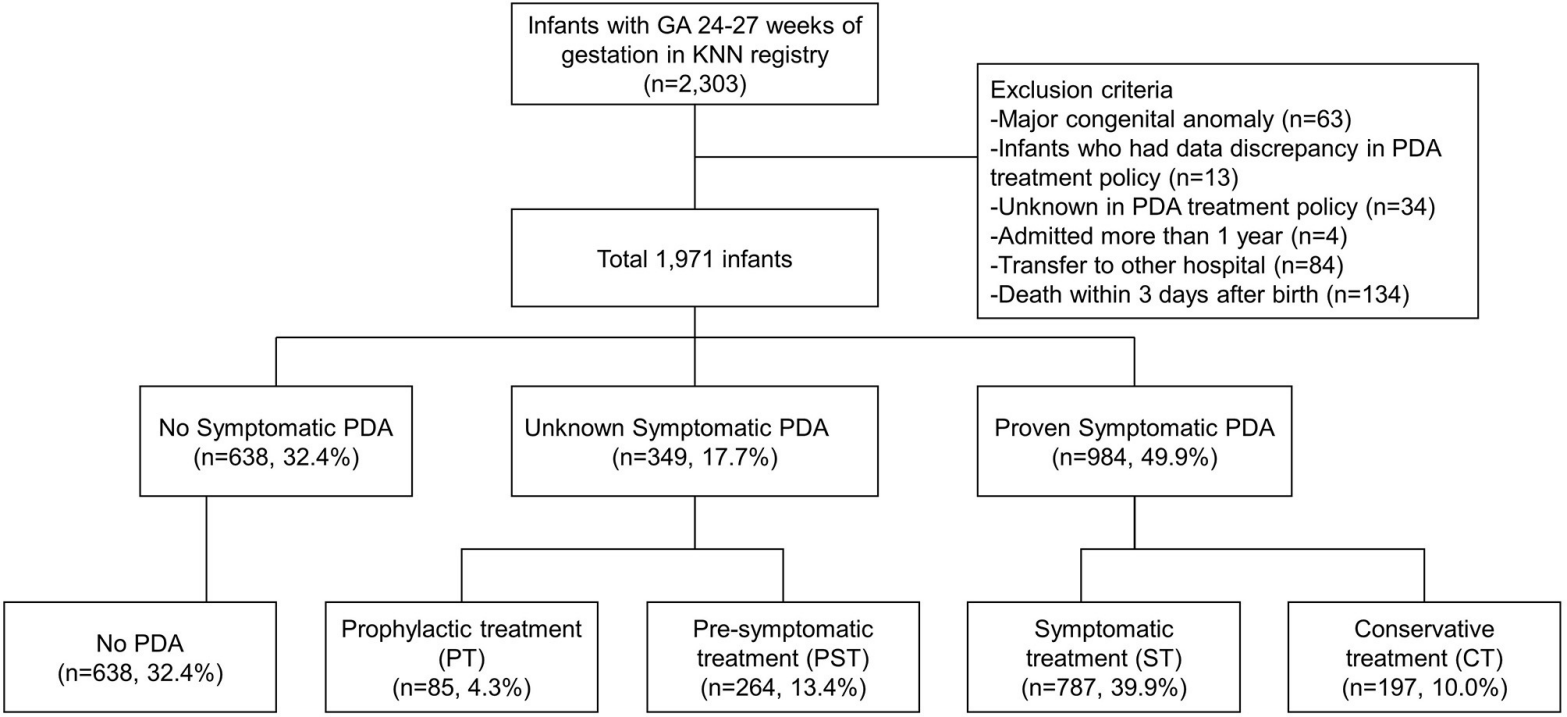
Flowchart of infants included in this study.

A total of 2,303 neonates born at 24–27 weeks of gestational age (GA) between 2013 and 2015 were identified from the KNN database (Figure 1). After exclusions, 1,971 infants with data on the presence or absence of PDA-related symptoms and outcomes were analyzed. Of them, 638 (32.4%) infants had PDA spontaneously closed without any symptom or sign of PDA. The remaining 1,333 (67.6%) infants were divided into the following groups according to therapeutic strategies used for PDA: PT group (85 infants, 4.3%), PST group (264, 13.4%), ST group (787, 39.9%), and CT group (197, 10%).

In the PT and PST groups, it was not known whether there were any symptoms or signs related to PDAs. In 984 infants with proven symptomatic PDA, 787 (80.0%) infants (ST group) received pharmacological or surgical intervention for PDA, while 197 (20.0%) infants (CT group) received conservative treatment without any intervention.

Results of comparison of clinical characteristics of infants according to groups are shown in Table 1. Gestational age, birth weight, multiple gestation, use of antenatal steroids, presence of histologic chorioamnionitis, DR resuscitation, 5-min Apgar, CRIB-II score, and early sepsis were significantly different among treatment groups. Infants in the ST group were more mature (GA: 26 [25, 27] weeks vs. 25 [24, 26] weeks, *p*_corrected_ < 0.001), heavier (birth weight: 840 g [705, 980] vs. 700 g [600, 890], *p*_corrected_ < 0.001), having less multiple gestation (31.6 vs. 42.6%, *p*_corrected_ = 0.021), and having less often use of antenatal steroids and less CRIB-II score than those in the CT group (*p*_corrected_ < 0.001).

**Table 1:** Clinical characteristics of subjects in different PDA treatment groups.

	**PT** **(*n* = 85, 4.3%)**	**PST** **(*n* = 264, 13.4%)**	**ST** ** (*n* = 787, 39.9%)**	**CT** **(*n* = 197, 10.0%)**	***p*-value**
Gestational age, weeks	25 [24, 26]	25 [25, 27]	26 [25, 27]^*^	25 [24, 26]	<0.001
Birth weight, g	790 [648, 950]	820 [671, 970]	840 [705, 980]^*^	700 [600, 890]	<0.001
Male	48 (56.5%)	133 (50.4%)	393 (49.9%)	102 (51.8%)	0.252
Multiple gestation	30 (35.3%)	65 (24.6%)	249 (31.6%)^*^	84 (42.6%)	0.001
Cesarean section	59 (69.4%)	186 (70.5%)	534 (67.9%)	146 (74.1%)	0.427
Antenatal steroid, complete	42 (49.4%)	114 (43.2%)	311 (39.5%)^*^	106 (53.8%)	0.002
SGA	8 (9.4%)	29 (11.0%)	80 (10.2%)	28 (14.2%)	0.573
PIH	9 (10.6%)	28 (10.6%)	66 (8.4%)	18 (9.1%)	0.814
Histologic CA	33/71 (46.5%)	83/241 (34.4%)	303/638 (47.5%)	88/178 (49.4%)	0.001
DR-resuscitation	3/83 (3.6%)	6/261 (2.3%)	62/765 (8.1%)	14/197 (7.1%)	0.006
5-min Apgar					<0.001
0–3	24/84 (28.6%)	23/261 (8.8%)	95/781 (12.2%)	20/196 (10.2%)	
4–6	37/84 (44.0%)	106/261 (40.6%)	344/781 (44.0%)	73/196 (37.2%)	
7–10	23/84 (27.4%)	132/261 (50.6%)	342/781 (43.8%)	103/196 (52.6%)	
CRIB-II score	13 [11, 15]	12 [10, 14]	12 [10, 14]^*^	14 [11, 16]	<0.001
Surfactant use	84 (98.8%)	261 (98.9%)	782 (99.4%)	197 (100%)	0.062
Early sepsis (<7 days)	1 (1.2%)	17 (6.4%)	65 (8.3%)	22 (11.2%)	0.048

*PT, prophylactic treatment; PST, pre-symptomatic treatment; ST, symptomatic treatment; CT, conservative treatment; SGA, small for gestational age; PIH, pregnancy-induced hypertension; CA, chorioamnionitis; DR, delivery room; CRIB, Clinical Risk Index for Babies*.

*p-values are results from Kruskal–Wallis test, chi-square test, or Fisher's exact test of all groups*.

* *p < 0.05 when compared with the CT group after Bonferroni correction*.

In-hospital neonatal mortality in the CT group was significantly higher than that in the PST or ST group (37.6 vs. 19.7% or 17.4%, both *p*_corrected_ < 0.001) (Table 2). The ST group had a higher percentage of those needing respiratory support at discharge (16.3 vs. 5.6%, *p*_corrected_ < 0.001), a higher incidence of BPD (61.8 vs. 46.2%, *p*_corrected_ = 0.005), and a higher percentage of those with ROP requiring treatment (33.9 vs. 21.8%, *p*_corrected_ = 0.011) than the CT group. However, there was no significant difference in the incidence of severe BPD between ST and CT groups (41.3 vs. 39.4%, *p*_corrected_ = 1.000). In multivariable logistic regression analysis after adjusting for baseline characteristics, in-hospital mortality and morbidities were related to PDA treatment strategies (Table 3).

**Table 2:** In-hospital outcomes of subjects in different PDA treatment groups.

	**PT** **(*n* = 85, 4.3%)**	**PST** **(*n* = 264, 13.4%)**	**ST** ** (*n* = 787, 39.9%)**	**CT** **(*n* = 197, 10.0%)**	***p*-value**
Mortality	37 (43.5%)	52 (19.7%)^*^	137 (17.4%)^*^	74 (37.6%)	<0.001
<1 month (28 days)	22 (25.9%)	29 (11.0%)^*^	75 (9.5%)^*^	56 (28.4%)	<0.001
Duration of invasive ventilation	21 [8, 43]	28 [11, 46]	29 [11, 49]	26 [8, 42]	<0.001
Duration of respiratory support	49 [22, 74]	67 [40, 94]^*^	70 [47, 96]^*^	54 [19, 83]	<0.001
Discharge with respiratory support	4 (4.7%)	25 (9.5%)	128 (16.3%)^*^	11 (5.6%)	<0.001
BPD at 36 weeks	27/54 (50.0%)	122/219 (55.7%)	413/668 (61.8%)^*^	61/132 (46.2%)	<0.001
Severe BPD	13/54 (24.1%)	85/219 (38.8%)	276/668 (41.3%)	52/132 (39.4%)	<0.001
NEC ≥ stage 2	6/84 (7.2%)	33/264 (12.5%)	99/787 (12.6%)	30/195 (15.4%)	0.100
IVH ≥ grade 3	17/85 (20.0%)	45/264 (17.0%)	181/787 (23.0%)	42/197 (21.3%)	0.013
PVL	5/78 (6.4%)	25/258 (9.7%)	95/779 (12.2%)	28/190 (14.7%)	0.203
ROP with surgery or VEGF	25/72 (34.7%)	69/228 (30.3%)	232/684 (33.9%)^*^	39/179 (21.8%)	<0.001
Hospital day	81 [23, 102]	96 [72, 117]^*^	95 [72, 116]^*^	90 [24, 121]	<0.001
Secondary PDA ligation	11 (12.9%)	58 (22.0%)	253 (32.1%)	0	<0.001

*PT, prophylactic treatment; PST, pre-symptomatic treatment; ST, symptomatic treatment; CT, conservative treatment; BPD, bronchopulmonary dysplasia; NEC, necrotizing enterocolitis; IVH, intraventricular hemorrhage; PVL, periventricular leukomalacia; ROP, retinopathy of prematurity; VEGF, vascular endothelial growth factor; PDA, patent ductus arteriosus*.

*p-values are results from Kruskal–Wallis test, chi-square test, or Fisher's exact test of all groups*.

* *p < 0.05 when compared with the CT group after Bonferroni correction*.

**Table 3:** Comparisons of outcomes among PDA treatment groups in multivariable logistic regression analysis.

	**PT vs. CT**	**PST vs. CT**	**ST vs. CT**
Mortality	1.614 (0.867, 3.005)	0.507 (0.311, 0.826)	0.349 (0.230, 0.529)
<1 month (28 days)	1.010 (0.519, 1.964)	0.350 (0.197, 0.620)	0.297 (0.186, 0.473)
BPD at 36 weeks	2.150 (1.002, 4.615)	2.208 (1.348, 3.616)	2.600 (1.679, 4.024)
Severe	0.745 (0.332, 1.671)	1.521 (0.924, 2.504)	1.413 (0.912, 2.191)
IVH ≥ grade 3	0.911 (0.456, 1.821)	0.836 (0.505, 1.385)	1.082 (0.712, 1.644)
PVL	0.413 (0.136, 1.252)	0.746 (0.400, 1.392)	0.949 (0.571, 1.576)
NEC ≥ stage 2	0.167 (0.038, 0.730)	0.808 (0.446, 1.466)	0.853 (0.516, 1.410)
ROP with surgery or VEGF	2.444 (1.218, 4.904)	2.587 (1.542, 4.341)	3.559 (2.263, 5.597)
BPD or death	2.036 (1.034, 4.007)	1.533 (0.983, 2.390)	1.704 (1.155, 2.514)
Severe BPD or death	0.976 (0.526, 1.810)	1.030 (0.668, 1.587)	0.901 (0.618, 1.312)

*PT, prophylactic treatment; PST, pre-symptomatic treatment; ST, symptomatic treatment; CT, conservative treatment; BPD, bronchopulmonary dysplasia; IVH, intraventricular hemorrhage; PVL, periventricular leukomalacia; NEC, necrotizing enterocolitis; ROP, retinopathy of prematurity; VEGF, vascular endothelial growth factor*.

*Data are shown as adjusted odds ratio (95% confidential interval) by binary logistic regression analysis adjusted for gestational age, multiple gestation, antenatal steroid complete, histologic chorioamnionitis, and delivery room resuscitation*.

The risk of death was significantly decreased in the PST group (aOR: 0.507; 95% CI: 0.311–0.826) and the ST group (aOR: 0.349; 95% CI: 0.230–0.529) compared with the CT group. The risk of composite outcome of BPD or death was significantly increased in the ST group (aOR: 1.704; 95% CI: 1.155–2.514), but not in the PST group, compared with the CT group due to an increased risk of BPD in the ST group. More importantly, the number of cases with severe BPD was not significantly increased in the PST group or the ST group compared with the CT group, although the risk of death was decreased in the PST group and the ST group compared to that in the CT group. Hence, the risk of composite outcome of severe BPD or death was not increased in the ST group or the PST group compared with the CT group.

Regarding morbidities, odds of BPD and ROP were significantly increased in PT, PST, and ST groups than in the CT group. Risks of severe BPD, IVH, and PVL in PT, PST, and ST groups were not significantly different from those in the CT group.

Regarding causes of neonatal death by treatment group, death due to pulmonary hemorrhage or neurological disease in the CT group was significantly higher than that in the PST group or the ST group (pulmonary hemorrhage: 5.1 vs. 0.8% or 1.3%, *p*_corrected_ = 0.024 or 0.014; neurologic disease: 6.1 vs. 1.5% or 1.0%, *p*_corrected_ = 0.048 or *p*_corrected_ < 0.001; Table 4).

**Table 4:** Causes of neonatal death according to treatment.

	**PT** **(*n* = 85)**	**PST** **(*n* = 264)**	**ST** **(*n* = 787)**	**CT** **(*n* = 197)**	***p*-value**
Neonatal mortality	22 (25.9%)	29 (11.0%)^*^	75 (9.5%)^*^	56 (28.4%)	<0.001
Cardiorespiratory	11 (12.9%)	10 (3.8%)^*^	31 (3.9%)^*^	24 (12.2%)	<0.001
RDS	0 (0.0%)	1 (0.4%)	6 (0.8%)	1 (0.5%)	0.773
PPHN	2 (2.4%)	2 (0.8%)	5 (0.6%)	4 (2.0%)	0.171
Pulmonary hemorrhage	4 (4.7%)	2 (0.8%)^*^	10 (1.3%)^*^	10 (5.1%)	0.001
Air leak	3 (3.5%)	2 (0.8%)	3 (0.4%)	2 (1.0%)	0.015
Others	2 (2.4%)	3 (1.1%)	7 (0.9%)^*^	7 (3.6%)	0.019
Neurologic	0 (0.0%)	4 (1.5%)^*^	8 (1.0%)^*^	12 (6.1%)	<0.001
Infection	3 (3.5%)	7 (2.7%)	17 (2.2%)	8 (4.1%)	0.470
GI problem	4 (4.7%)	6 (2.3%)	12 (1.5%)	5 (2.5%)	0.209
Others	4 (4.7%)	2 (0.8%)^*^	7 (0.9%)^*^	7 (3.6%)	0.005

*PT, prophylactic treatment; PST, pre-symptomatic treatment; ST, symptomatic treatment; CT, conservative treatment; RDS, respiratory distress syndrome of newborn; PPHN, persistent pulmonary hypertension of newborn; GI, gastrointestinal*

*p-value are results from chi-square test or Fisher's exact test of all groups*.

* *p < 0.05 when compared with the CT group after Bonferroni correction*.

## Discussion

Optimal therapeutic strategies for PDA in extremely preterm infants born at or before 28 weeks of gestation who are at high risk of hsPDA remain uncertain. Some retrospective cohort studies have reported that a conservative treatment for symptomatic PDA could reduce the morbidity of pharmacological or surgical therapies without increasing consequences of persistent ductal patency. Abandoning routine interventions and/or more selective individualized interventions to close PDA in preterm infants has been recommended (17, 18, 28). However, there are a few exceptions according to these studies. That is, rescue pharmacological or surgical intervention to close the PDA is allowed for infants in conservative treatment groups if they meet rescue criteria such as pre-specified respiratory and hemodynamic criteria of hsPDA.

Taking a step forward about a conservative treatment for PDA, some studies (19–21, 29, 30) have performed conservative treatment alone without using any pharmacological or surgical intervention for symptomatic PDA as a rescue management, just waiting for spontaneous PDA closure in extremely preterm infants with hsPDA, although there is no sufficient evidence from large randomized clinical trials about its benefits for neonatal outcomes. Its potential side effects are unknown either. These trials using non-interventional conservative treatment might have been attributed to an understanding of the natural course of PDA and outcomes of preterm infants without receiving any pharmacological or surgical intervention for symptomatic PDA. Actually, a large proportion of extremely preterm infants show spontaneous closure of PDA with only conservative management without any intervention for PDA closure before discharge (30).

However, even if PDA is spontaneously closed without any intervention someday, prolonged exposure to hsPDA in extremely preterm infants who have a high risk of PDA-related morbidities might potentially increase their morbidities and mortality.

A retrospective time series observational cohort study (22) found that a non-interventional conservative approach for PDA closure was not associated with an increased in-hospital mortality compared with a mandatory PDA closure approach. Although it showed a very low mortality compared to other centers, the mandatory approach included aggressive surgical intervention for 82% of infants in early neonatal periods. Another historical control study (31) has compared two cohorts of infants in whom PDA is managed differently and found that completely withholding PDA treatment is not associated with increased mortality or morbidities in VLBW infants. However, these non-controlled historical observational studies cannot ascertain the true relationship between changing therapeutic strategies for PDA and changes in their better outcomes (32). Therefore, the safety and possible efficacy of non-interventional conservative treatment for hsPDA cannot be generalized with only two single-center studies.

In a network meta-analysis (33) of 68 randomized trials, the mortality rate was 11.9% in all studied groups and 17.4% in the non-pharmacological treatment group. The placebo group without treatment did not show higher risk of mortality, NEC, or IVH compared to groups with other pharmacological treatments, although the placebo group ranked the worst in terms of PDA closure rate.

Another network meta-analysis (34) of 24 observational studies in addition to RCT reported that active pharmacological treatment was superior to non-pharmacological treatment in decreasing risks of unfavorable clinical conditions associated with PDA. By decreasing the need for surgical interventions, effective pharmacological treatment can avoid surgical adverse effects and complications. However, the non-pharmacological treatment group had similar mortality and risks for overall adverse effects to groups with active pharmacological treatments, although the non-pharmacological treatment group had a higher rate of failure to close PDA. Such lack of improvement for neonatal outcomes in groups with active pharmacological treatments for PDA in these network meta-analyses might be due to several various limitations such as confounding factors and/or bias of clinical trials (28). These analyses seemed to illustrate the concept that a non-interventional conservative treatment for hsPDA does not appear to lead to increased morbidity or mortality compared with other therapeutic strategies for PDA in preterm infants. However, they did not reveal better outcomes of a non-interventional conservative treatment compared with other therapeutic strategies for PDA in preterm infants.

Although the anticipated benefit of a non-interventional conservative treatment to reduce possibilities of morbidities and mortality related to interventions seems to be promising, there is no sufficient evidence that can justify not using a rescue management through pharmacological or surgical interventions for PDA with features of clinical compromise attributable to hsPDA.

In our study, among infants with proven symptomatic PDA, 20% of infants who were managed with conservative treatment without any intervention had higher mortality than those who received symptomatic treatment. In multivariable logistic regression analysis after adjusting for baseline characteristics, their risk of mortalities was significantly increased than those who received symptomatic treatment through pharmacological or surgical interventions.

Although the ST group showed significantly increased odds of BPD at 36 weeks of GA and combined outcome of BPD and death, severe BPD and combined outcome of severe BPD and death were not significantly different between the ST group and the CT group. These observations suggest that increases in other morbidities such as BPD and ROP may reflect increasing survival of extremely preterm infants who receive symptomatic treatment.

Our data are in agreement with another retrospective multicenter cohort study of preterm infants with birth weight < 1,000 g in Brazil (29). Among infants with echocardiographic diagnosed PDA, regardless of the presence of PDA symptoms, 37.8% of infants were managed with conservative treatment without any pharmacological or surgical intervention. Although conservative and pharmacological treatments were protective for the combined outcome death and BPD, the conservative treatment group had higher mortality than the group receiving pharmacological or surgical intervention.

In our study about main causes of neonatal death according to treatment strategies, the incidence of pulmonary hemorrhage in those with cardiorespiratory causes and neurological causes involving IVH was significantly higher in the CT group than in the ST group. By abandoning rescue management for hsPDA through non-interventional conservative treatment, prolonged exposure to hsPDA might increase PDA-related morbidities such as pulmonary hemorrhage and intraventricular hemorrhage, consequently leading to death.

### Limitations

This study has some limitations. First, it lacked detailed echocardiographic data and definite periods of initiation of PDA treatment in preterm infants with symptomatic PDA. In addition, information about primary treatment methods of treatment groups (whether, it was pharmacological or surgical treatment) was insufficient. However, data were analyzed by classifying treatment strategies for individual patients entered by local staffs according to the manual of operation, although it was a cohort study. Therefore, we could compare a symptomatic, conservative treatment group with PDA treatment groups. Furthermore, although medical records were analyzed by researchers at each hospital to minimize bias in relation to determining primary causes of death, sometimes it was not easy to distinguish one major cause of death for those with complex conditions where multiple causes interacted with each other.

## Conclusions

Our nationwide cohort data showed that a considerable number of Korean extremely preterm infants with proven symptomatic PDA received only conservative treatment without any pharmacological or surgical interventions in the lower gestational age and lower birth weight group. They had significantly higher mortality due to pulmonary hemorrhage and intracerebral hemorrhage than those who received symptomatic treatment. Our results also showed that symptomatic treatment with pharmacological or surgical intervention for those with proven symptomatic PDA might increase their survival without increasing severe BPD.

For extremely preterm infants who are at highest risk of PDA-related morbidities and mortality, although a less interventional approach for PDA can be allowed, rescued pharmacological or surgical interventions may be considered if they meet rescue criteria for hsPDA.

Future randomized controlled trials comparing short-term outcome and long-term neurodevelopmental outcome between no interventional conservative approach and other less interventional selective approaches with rescue management are warranted.

## Data Availability Statement

Data availability was subjected to the Act on Bioethics and Safety [Article 18 (Provision of Personal Information)] of Korea. Data sharing or data access can be possible only through the data committee of Korean neonatal network (http://knn.or.kr) and after obtaining permission from the Korea Centers for Disease Control and Prevention. Detail contact information was as follows: Data Access Committee: Yun Sil Chang (cys. chang@samsung.com) and Ethics Committee: Jang Hoon Lee (neopedlee@gmail.com).

## Ethics Statement

The studies involving human participants were reviewed and approved by Korean Neonatal Network registry. Written informed consent to participate in this study was provided by the participants' legal guardian/next of kin.

## Author Contributions

JS and JL conceptualized and designed the study, drafted the initial manuscript, and reviewed and revised the manuscript. SO and EL designed the data collection instruments, collected data, carried out the initial analyses, and reviewed and revised the manuscript. BC conceptualized and designed the study, coordinated and supervised data collection, and critically reviewed the manuscript for important intellectual content. All authors approved the final manuscript as submitted and agree to be accountable for all aspects of the work.

## Funding

This work was supported by the Research Program funded by the Korea National Institute of Health (2019-ER7103-01).

## Conflict of Interest

The authors declare that the research was conducted in the absence of any commercial or financial relationships that could be construed as a potential conflict of interest.

## Publisher's Note

All claims expressed in this article are solely those of the authors and do not necessarily represent those of their affiliated organizations, or those of the publisher, the editors and the reviewers. Any product that may be evaluated in this article, or claim that may be made by its manufacturer, is not guaranteed or endorsed by the publisher.
